# Multi-Morbid Health Profiles and Specialty Healthcare Service Use: A Moderating Role of Poverty

**DOI:** 10.3390/ijerph16111956

**Published:** 2019-06-01

**Authors:** Ilan Kwon, Oejin Shin, Sojung Park, Goeun Kwon

**Affiliations:** 1 School of Social Work at Michigan State University, Baker Hall, 655 Auditorium Road, East Lansing, MI 48824, USA; 2 School of Social Work at University of Illinois Urbana-Champaign, 1010 W Nevada St, Urbana, IL 61801, USA; oeshin2@illinois.edu; 3 Brown School of Social Work at Washington University, 1 Brookings Drive, Saint Louis, MO 63130, USA; spark30@wustl.edu (S.P.); g.kwon@wustl.edu (G.K.)

**Keywords:** multi-morbidity, health profiles of older adults, specialty healthcare, poverty

## Abstract

Increasing life expectancy in the USA makes a better understanding of the heterogeneous healthcare needs of the aging population imperative. Many aging studies have discovered multimorbid health problems focusing mainly on various physical health conditions, but not on combined mental or behavioral health problems. There is also a paucity of studies with older adults who use professional healthcare services caring for their mental and substance-related conditions. This study aims to enhance the knowledge of older peoples’ complex healthcare needs involving physical, mental, and behavioral conditions; examine the relationship between multi-morbid health profiles and specialty healthcare service utilization; and investigate its association to poverty. The study data were derived from the National Survey on Drug Use and Health (NSDUH) in 2013 (*n* = 6296 respondents aged 50 years and older). To identify overall health conditions, nine indicators, including physical, mental, and substance/alcohol, were included. Healthcare service utilization was measured with four mutually exclusive categories: No treatment, mental health treatment only, substance use treatment only, and both. We identified four health profiles: Healthy (82%), having physical health problems (6%), physical and mental health problems (4%), and behavioral problems (8%). Older people’s health profiles were differentially associated with healthcare use. Those living in poverty with both physical and mental health problems or substance/alcohol health problems were less likely to receive mental health and substance use treatments than those with more financial resources. Implications for geriatric healthcare practices and policy are discussed.

## 1. Introduction

Although older people comprise about 13% of the US population, they accounted for 34% of healthcare-related spending in 2010 [1]. Average medical spending per capita in 2015 was $59,000, which accounts for 16.8% of all spending among those aged 65 and over and 6.7% of spending overall [2]. One-third of the middle-aged (between 45 and 65 years) and about two-thirds of the older age group (61% of people aged 65 years or more) reported having more than two health conditions [3]; the prevalence of multi-morbidity in the older population has increased from 55.4% in 2002 to 61.6% in 2015 [4].

To provide cost- and outcome-effective healthcare services, a primary task is to make an accurate diagnosis of older individuals with multiple healthcare needs. However, many studies on multi-morbidity tend to focus on physical health conditions, such as high cholesterol, high blood pressure, heart disease, stroke, and diabetes [1,5,6]. Given that depression is the fourth leading cause of death among adults age 65 and over [7] and one in five adults aged 55 or older reports experiencing mental health problems, such as depression and anxiety [8], the inclusion of mental health conditions among older adults will increase their multi-morbidity rates. In addition, substance misuse is another health concern with aging baby boomers [9]. Approximately 6% to 16% of older adults are considered at risk of heavy drinking [9], which can cause serious health problems, such as cancer, liver damage, and brain damage, as well as worsen health conditions, such as diabetes, high blood pressure, stroke, and even mood disorders [10]. 

Some studies investigated specific combinations of health conditions among older adults. For example, physical–mental co-morbidity in older adults is common, in particular among those with a higher number of physical disorders [11] and those with both substance/alcohol abuse and mental health problems [12]. A strong association between mental health problems and alcohol/substance abuse among older adults is well known [13,14]. According to a Substance Abuse and Mental Health Services Administration’s report [15], approximately 20% of older adults who have mental illness meet the criteria for substance dependence or abuse, and about 40% of those with substance dependence or abuse issues also report having mental illness. Some studies argue that alcohol use disorder (abuse and dependence) should be examined because the prevalence of risky drinking behaviors among older individuals is high [9], but their drinking patterns are less likely to be detected by clinicians [16]. 

Further, recent studies have explored multi-morbid health conditions among older individuals by using latent clustering analyses (LCA). For example, Schüz, Wurm, Warner, and Tesch-Römer [17] explored age-related health profiles among people aged 40 to 85, identifying four groups: Individuals with no disease, cardiovascular disease, joint disease, or multiple illnesses. In another study, community-dwelling adults aged 64 and more were categorized into four groups: People who are physically impaired, cognitively and physically impaired, cognitively impaired, and relatively healthy; only about one-third were in the healthy group [18]. Liu, Tian, and Yao [19] emphasized older adults’ functioning level with four identified groups: Frail, functionally impaired, highly co-morbid, and relatively healthy. Focusing on older people’s health profiles associated with marital status and living arrangements, Park, Smith, Dunkle, Ingersoll-Dayton, and Antoucci [20] revealed that older individuals with multiple health conditions who live alone are more likely to suffer from depression. 

Despite the increasing older population with multi-morbidity, little attention has been paid to whether they receive appropriate healthcare services, especially for mental health and substance abuse problems. When older individuals have mental health or substance abuse problems, they are more likely to visit primary care physicians than to seek professional healthcare services [21,22]. In general, one-third of older adults reported they did not receive mental health or substance abuse treatments although they perceived they needed such treatment [7,8]. According to a recent national report [23], among those aged 50 years or older with a mental illness, only one-fifth used mental health services; among those with substance abuse or dependence, only about 10% received treatment at a specialty healthcare facility. Park-Lee et al. [23] also reported that more than half of older adults (51.9%) with both mental health and substance abuse problems did not receive any mental health or substance abuse treatment at a specialty healthcare facility. Considering the strong association between mental health and substance abuse problems [24], the provision of professional healthcare services is critical. 

Research has reported that the lack of specialty healthcare service utilization is particularly true among older men [25,26], members of underrepresented racial and ethnic groups [27,28], and those with lower incomes [29,30]. The inability to afford the cost of care was the most commonly reported reason for not receiving mental health [23] and substance abuse treatment (along with a lack of readiness to stop using the substances) [31]. Gum, Iser, and Petkus [32] showed that older adults of lower socioeconomic status (SES) were less likely to receive behavioral health services.

The burden of healthcare costs can be critical among poor older individuals with multi-morbidity. They may need to visit healthcare services more frequently, use a greater variety of services to meet their complex healthcare needs, and take more medications. Although Medicare and other public supplemental insurance largely cover their healthcare utilization costs, out-of-pocket spending increases with the number of health conditions. Compared to persons without any health problems, the average annual out-of-pocket expenditure was 3.7 times more for those with two chronic health conditions, 4.8 times more for those with three conditions, and 7.5 times more for those with five health conditions; $216 for people without chronic health conditions versus $1622 for those with five conditions [33,34]. Medicare beneficiaries may have a deductible or pay-a-monthly premium for additional services. Older patients with multi-morbidity make use of more services than those without at greater cost [35]. Given that the low rate of older adults’ healthcare service use is associated with low income [36], older adults who live in poverty with multi-morbid health problems are expected to experience a greater out-of-pocket burden for healthcare and will be deterred from utilizing professional healthcare services. 

To examine the relationship between older adults’ health, their healthcare service use patterns, and the moderating role of poverty, we used the health behavioral model [37], which posits that service use can be determined by predisposing factors that exist before the onset of symptoms or illness (age, gender, and education); enabling factors, including social and financial; or need factors, like perceived health problems. Also, we applied some life course perspectives that account for the influence of socioeconomic environments on health inequality in mid- and later life. According to stress process theory [38], people who have lower social and economic status are exposed to more lifetime stressors and have fewer resources to reduce their stressors over time, which influences physical and mental health outcomes in old age. Similarly, cumulative inequality theory [39] indicates that disadvantages accumulate, and both cumulative disadvantage and the stress process result in cumulative inequality. Based on the previous literature and these theoretical backgrounds, the current study examines the following research questions:

(1) What are the patterns of multi-morbidity (physical, mental, and substance/alcohol problems) among older adults in the United States? We expect to identify several heterogeneous groups based on their physical, mental, and behavioral health conditions.
(2) What is the relationship between older adults’ multi-morbid health profiles and the patterns of use of specialty healthcare services? It is hypothesized that older adults with mental or behavioral health problems use more mental health and/or substance use services.
(3) To what extent does poverty moderate the relationship between health profiles and patterns of specialty health service use? It is expected that those living in poverty have limited access to healthcare treatments despite their mental or behavioral problems.

## 2. Materials and Methods 

### 2.1. Data and Sample

This study used data from the 2013 wave of the National Survey on Drug Use and Health (NSDUH), a nationally representative survey that provides information on health and the use of illegal drugs among the US population aged 12 or older. Questions regarding mental health problems, mental health treatment, and other health-related behaviors are also included [13,40]. Data collection is based on a multistage complex survey design: The first selection is by census tract, the second by geographic area, the third by dwelling unit, and the last the individuals, oversampling in the eight largest states in the US [40].

The sample size varied in the extent of which variables were used for the analytical models. After dropping all missing values, a total of 6325 responses was used for running LCA with nine health indicators, 6294 for conducting multinomial logistic regression to examine the characteristics of each health profile group, and 6278 for examining the relationships among the health profiles, poverty, and specialty healthcare service use. 

### 2.2. Measures

Health conditions. Physical health was measured by whether respondents had multiple chronic conditions (MCCs; diabetes, heart disease, high blood pressure, lung cancer, stroke, and ulcer), self-rated overall health, and functioning in daily activities. Among several physical health problems asked in the NSDUH dataset, six diseases appertaining to MCCs were selected by the most common chronic conditions among older adults that the US Department of Health and Human Services identified [41]. Respondents having more than two health conditions were coded 1 for MCCs, or else 0. Self-rated health was coded excellent, very good, good, fair, and poor in a binary variable (0 = excellent/very good/good and 1 = fair/poor). Functioning in daily activities was measured by the WHO Disability Assessment Schedule (WHODAS), a widespread measure of disability and functional impairment in six domains: Cognition, mobility, self-care, getting along with others, life activities, and participation in society [42]. The NSDUH revised WHODAS into 8 levels based on the severity of overall functioning, which was recoded as a binary variable in this study (0 = no functional impairment, 1 = functional impairment). Mental health was measured by the level of severe psychological distress, the diagnosed index of depression and anxiety, and suicide thought. Psychological distress was assessed by the short-version of the Kessler scale: (a) Feeling nervous, (b) hopeless, (c) restless or fidgety, (d) so sad or depressed that nothing could cheer them up, (e) everything was an effort, and (f) down on oneself [43]. The original scale ranges from 0 to 24, with a higher score indicating more psychological distress. It was coded as a binary variable (scores less than 12 coded 0 = no serious psychological distress, scores over 12 coded 1 = scores for serious psychological distress). The NSDUH survey also asked whether the respondent experienced depression and/or anxiety in the past year and whether he/she seriously thought about suicide (0 = no and 1 = yes). Respondents were asked if they had been told they had alcohol use disorder (AUD) and substance use disorder (SUD) in the past year. Their answer was coded as a binary variable, 0 = no alcohol/substance use disorder; 1 = other. 

Specialty healthcare service use. Since the literature indicated a lack of professional healthcare service utilization in the older population [21,22], the study aims to examine if older people receive specialty healthcare services, indicating professional mental health or substance-related treatments rather than treatments or services provided in primary care, when they need. Types of service utilization in this study were constructed with four mutually exclusive categories: No treatment, mental health treatment only, substance use treatment only, and both treatments. For mental health, respondents were asked if they received inpatient or outpatient mental health treatment or medication for mental health treatment in the past year. The NSDUH dataset created a single dichotomous variable; if respondents reported having at least one type of mental health treatment (inpatient, outpatient, or medication), the variable was coded 1; if not, it was coded 0. Treatment for substance use disorder was assessed in a similar way. The survey asked if respondents received any treatments for alcohol/substance abuse or dependence in the past year at various places, including a hospital, an inpatient or outpatient rehab facility, a mental health center, or a doctor’s office. Treatments at an emergency room, in a prison or jail, and a self-help group were excluded for this variable. Both questions, asking about alcohol or substance use treatments, were coded as binary variables: If respondents received at least one treatment, they were coded 1, or else they were coded 0. The dependent variable of healthcare service utilization was constructed with four categories: No treatment, mental health treatment only, substance use treatment only, and both mental health and substance use treatment.

Poverty. Given that an individual’s poverty level is strongly associated with their health service use [44,45], the poverty variable, rather than household income or socioeconomic status (SES), was used to examine its relationship with older adults’ specialty healthcare services use. The poverty variable was created in the NSDUH dataset along with the poverty threshold that the US Census Bureau determined based on age, family size, the number of children in the household, and total family income [46]. Individuals who reported their family income as the same as the poverty threshold indicated living at 100% of the poverty threshold or, in other words, living in poverty. In this study, respondents who lived in poverty were coded 1, or otherwise 0. 

Covariates. Some demographic variables were included as covariates. Age was coded into two groups: Aged 50 to 64 and aged 65 and above. Gender was dummy coded as 0 (men) or 1 (female). Marital status was coded as 0 (divorced, widowed, separated, or never married) or 1 (married); household composition was coded as 0 (living alone) or 1 (living with other family members), and insurance coverage was coded as 0 (services not fully covered) or 1 (fully covered). Some covariates were coded categorically. Race/ethnicity was coded: 0 = white, 1 = black, 2 = Hispanic, and 3 = other. Education was coded from 0 (less than high school) to 4 (college and above).

### 2.3. Analysis

We used latent class analysis (LCA) to identify groups of respondents based on the nine dichotomous health indicators. Latent class analysis (LCA) assumes that variables reflect meaningful latent characteristics [47]. To estimate the best LCA model, several steps were followed. First, a basic model with nine binary indicators was created, designating all indicators as one latent class. Second, the number of latent classes was increased until the best model was identified based on three model fit indices (Bayesian information criterion (BIC), classification quality entropy, and Lo–Mendell–Rubin adjusted likelihood ratio test) as well as the interpretability of the classes [48]. Classification quality entropy is a single value measure of the degree of uncertainty in the model, which indicates how well individuals are assigned to latent classes [47]. The entropy estimates range from 0 to 1 and values closer to 1 indicate better assignment. A better model reveals smaller BIC estimates, entropy closer to 1, and a *p* < 0.05 for the Lo–Mendell–Rubin adjusted likelihood test. In addition, a rule of thumb is that each latent class includes a minimum of 3% to 4% of the total cases. Mplus version 7.4 was used for the latent class analysis models, including weights and complex survey design variables [48]. 

We also conducted multinomial logistic regression analysis to examine the association between identified health conditions and the four categories of healthcare service utilization: No treatment at all, mental health treatment only, substance use treatment only, and both treatments. Multinomial logistic regression outcomes predict the effect of category membership on a dependent variable [49]. The main effects of health conditions (or health profiles) and healthcare service use were examined, controlling for poverty. To examine the moderating effect of poverty on the association between the health profiles and healthcare services’ utilization, an interaction term was created by multiplying the poverty variable with the health profiles and was added to the multinomial logistic regression model. These analyses were conducted using STATA version 14 (StataCorp LLC, College Station, TX, USA).

## 3. Results

**Figure 1:**
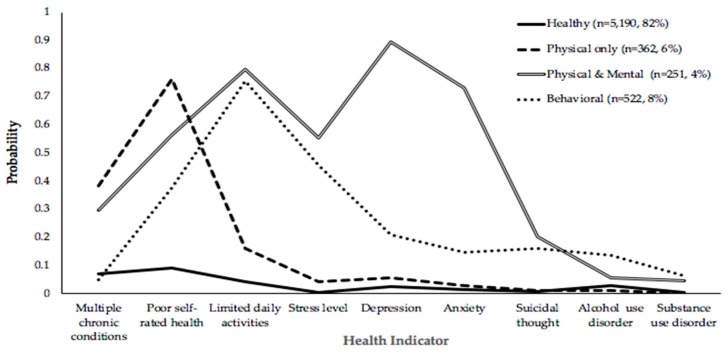
Class profiles of the health condition (*n* = 6325).

Table 1 shows the LCA results identifying the groups of participants’ health conditions. The four-class model was determined as the best fit to this data based on the smallest BIC value (BIC = 27,736.929), an entropy estimate close to 1 (entropy = 0.76), and a significant LMR test result (*p* = 0.001). The smallest proportion among the total four classes was 4% for older people with physical and mental health problems, followed by 6% for those with physical problems only, and 8% for those with substance/alcohol problems; the majority (82%) were healthy. To define and name each latent class, two methods were used. The first was based on test results: Conditional probabilities of 0.7 to 1 indicate a high probability of belonging to that class, 0.4 to 0.69 a moderate probability, and less than 0.4 a low probability [47,50]. A second method is to assign individuals to the latent class showing their highest probability of membership [51]. Taken together, the four groups were labeled as healthy, physical only, physical and mental, and behavioral—including both mental and substance/alcohol problems (Figure 1). 

Among the total sample, the largest group was healthy (82%), followed by having behavioral health problems (8%), physical health problems only (6%), and physical and mental health problems (4%). Older individuals with physical problems showed a high probability of a self-rated poor health condition and the highest probability of having MCCs. Those with both physical and mental health problems showed high probabilities of depression, anxiety, and limited functioning in daily activities, moderate probabilities of self-rated poor health and serious psychological distress, and the highest probability of suicide thoughts. The group with behavioral health problems showed a high probability of limited functioning in daily activities, a moderate probability of serious psychological distress, and the highest probability of substance/alcohol problems.

Multinomial logistic regression was used to assess sociodemographic predictors of membership in each class, with the healthy group as the reference category (Table 2). In general, among the 6294 participants, 74% were white, and 45% were male. Approximately, three-fifths were 50 to 64 years of age (62%) and married (59%). The majority graduated high school (85%), reported not living in poverty (89%), and had insurance that covered their healthcare service use (91%). One-fourth lived alone. Compared to those under 65, for those over 65, the relative risk of being in the group with physical health problems or the group with physical and mental health problems decreased by a factor of 0.26 and 0.41, respectively, whereas the relative risk ratio of being in the group with behavioral health problems increased by a factor of 1.48. Compared to white older adults, blacks were less likely to have physical health problems, but more likely to have behavioral health problems. Females were more likely to be in the groups of physical health problems or physical and mental health problems. Older adults living in poverty were more likely to be in one of the groups with health problems rather than being in the healthy group. 

Table 3 indicates the results of the multinomial logistic regression for identification of the main and interaction effects of older individuals’ health profiles and poverty on specialty healthcare service use. The groups with health problems were more likely to use specialty healthcare services than the healthy group. Older adults living in poverty were more likely than those not living in poverty to use mental health treatments (Relative Risk Ratio, RRR = 1.62, *p* = 0.02) and both mental health and substance use treatments (RRR = 3.86, *p* = 0.002). However, older individuals living in poverty with physical and mental health problems or a behavioral health problem were, respectively, 0.20 times or 0.31 times less likely to receive both treatments.

Beyond the key study variables, some demographic variables were significantly associated with the use of specific healthcare services. Those aged over 65 years were less likely to use any type of treatment than those aged between 50 and 64 years. Racial differences were significant only for mental health treatment; older adults of color were less likely to use mental health treatments than whites. Females were more likely to receive mental health treatment and males were more likely to receive substance use treatment. Married older people were less likely to receive substance use treatment. Older individuals with more education were more likely to use mental health treatment, but less likely to use substance use treatment. Compared to older individuals without full insurance coverage, those with full coverage were less likely to receive mental health treatment. 

## 4. Discussion

This study aimed to examine the patterns of multi-morbidity in the older population and their relationships with specialty healthcare service utilization as a function of age and poverty. Although many studies have focused on older people’s multi-morbidity with multiple physical diseases [5,6] or set comorbid psychiatric and substance abuse disorders apart from physical diseases [13,14], some recent studies intimate the increased proportion of community-dwelling older individuals with combined physical, mental health, or substance abuse problems and a need for caring for them in the public system of healthcare [52,53]. Consistent with the new approach, our findings make several important contributions to geriatric healthcare as well as the healthcare service delivery system. 

First, it is urgent to integrate the geriatric primary care system with the provision of mental and behavioral healthcare services. The findings highlight the different characteristics of multi-morbid health conditions among older adults. The older population was divided into four groups: Healthy, having physical health problems, physical and mental health problems, and behavioral (substance/alcohol) health problems. One third of people aged 45 to 64 years of age and more than half of those 65 and older have multiple chronic conditions (MCCs) [3,4]. Research indicates that older adults with MCCs perceive their overall health as worse than healthy people [54,55], and they also have an increased risk of mortality [56]. Older individuals who suffer from mental problems tend to perceive their overall health condition as poor [57], frequently show co-morbid physical health problems [11], and are often unable to effectively manage their life, causing limited everyday functionality [58]. 

Those 65 and older were more likely to be in the group with behavioral health problems than those under 65. It is noteworthy that the proportion of older individuals with behavioral health problems is higher than that for the other two non-healthy groups. In addition to substance/alcohol misuse problems, this group scored especially high on serious psychological distress, a symptom which has demonstrated adequate sensitivity and distinguish psychiatric diagnoses such as depression and anxiety that are categorized by the Diagnostic and Statistical Manual of Mental Disorders, 4^th^ edition [DSM-IV] in the older population [24,59]. The relatively high proportion of older adults with behavioral health problems is consistent with previous literature [12,14,31]. Some scholars warn that behavioral health problems may be underestimated due to misdiagnosis [9,16].

Nevertheless, the lack of mental health and substance use treatment is still a problem in the geriatric healthcare system [7,60,61,62]. Consistent with the literature [22], older adults are less likely to use special mental health and substance use services than relatively younger older adults. In particular, the study results are consistent with the previous literature showing minority older adults are less likely than whites to use mental health services [27,28], old men are less likely than women [26] as are people with lower education levels [29,30]. The integrated healthcare services in primary care may make older patients’ access to specific mental health or substance use treatments more straightforward than those treatments in professional and private care systems. 

Second, the study draws careful attention to the role of mental health conditions among older adults. As expected in Andersen’s model, older individuals’ healthcare service utilization is determined by their health conditions. In particular, if they perceive more healthcare needs, they are more likely to receive mental health or substance abuse treatments. An interesting result in our findings, however, is that older individuals with solely physical health problems were more likely to seek mental health treatment than healthy persons. Older patients with physical health conditions may not be diagnosed with mental health disorders, but they could perceive a need for mental healthcare [37]. A strong physical–mental co-morbidity has been confirmed [11], and some studies emphasize that mental health should be considered when diagnosing physical health problems in older people [63,64]. Older adults’ risk of having anxiety is elevated as the number of their medical conditions increases [65], and this co-morbidity complicates help-seeking, diagnosis, treatment, and prognosis [64]. Alcohol/substance abuse in old age is also related to social isolation, loneliness, chronic medical pain, and further impairment in daily functioning [53]. Mental health integration in the geriatric system of care will be an essential component of preventive medicine and chronic disease management for older patients with complex multi-morbid health problems. 

Finally, our findings emphasize the development of a comprehensive healthcare treatment system, especially for poor older adults with multi-morbidity. Common characteristics of older people in the three non-healthy profiles include living in poverty, less education, and being currently unmarried. These results are in accordance with the stress process theory [38] and cumulative inequality theory [39]. Older individuals with few financial and social resources might be more exposed to lifetime stressors, have more difficulties in overcoming their problems or obstacles, and result in more health problems throughout the cumulative disadvantages and distress. Several studies support such life course perspectives. For example, older adults who have low socioeconomic status (SES) are more likely to be depressed [66,67] and less likely to access services to care for their mental health challenges [68]. Some studies indicate that inequality in health is associated with race: African Americans, Hispanics, and people of low SES living in the United States are more susceptible to environmental stressors and show higher rates of physical health problems, such as hypertension, heart disease, and stroke, than whites and people of higher SES [69], and have more mental health problems [70].

Ironically, the study results showed that older adults living in poverty were more likely to use both mental health and substance use treatments than those with more resources, which is inconsistent with the previous literature. However, it may be possible that poor older adults use more medical goods and services because a more substantial portion of their expenses is paid for by the government, mostly Medicare [2]. Regarding insurance, older adults whose services were fully covered by their health insurance were less likely to use mental health treatment. Some studies suggest that the influence of poverty level on health service utilization must control for health insurance because out-of-pocket expenditures can influence poor older adults’ healthcare service utilization [45,47]. Use of a combined measure that accounts for poverty and health insurance coverage may untangle the association among poverty, health insurance, and healthcare service use in older adults. 

Older individuals who live in poverty with physical and mental health problems or behavioral health problems were less likely to receive either mental health or substance use treatment. Although federal health insurance, such as Medicare, pays for 65% of older people’s medical expenses and about 13% is paid by private insurance, nearly 20% of payments are out-of-pocket [2]. Among older individuals living in poverty, out-of-pocket expenditure is a high burden regardless of the insurance type [71,72]. Some studies in the framework of cumulative inequality theory highlight that older individuals who are living in extreme poverty are much less likely to be healthy and to access medical resources [45], and ascribe the inequality in healthcare services to a lack of clarity about the target population of integrated care approaches [73]. Future research and healthcare policy need to manifest this prevailing inequality and suggest the need for social justice and health service equity for the most disadvantaged older adults who need to visit several different healthcare systems to care for their complex health problems. 

The present findings should be interpreted with some caution. First, the cross-sectional nature of our data precludes drawing causal inferences related to the association reported. Second, mental health and alcohol or substance use behaviors were respondents’ self-reports, which are subject to a variety of biases associated with memory errors and underreporting [74]. In addition, individuals who were institutionalized or homeless were not included in the data sampling; some settings may contain a higher frequency of substance use than is typically found in the community [75]. Finally, the data provides variables for receiving mental health and substance use treatment, but not primary care. 

## 5. Conclusions

Despite these limitations, this study provides new information for geriatric healthcare practice and policy, and points toward future research. The primary need is an integrated healthcare system that includes mental and behavioral healthcare with geriatric primary care. Mental health integration is an essential component of preventive medicine and chronic disease management for older patients [76]. Improved screening of older adults’ heavy or binge drinking behaviors, or even a single behavioral intervention, can prevent negative alcohol–medication interactions [9] and improve health outcomes [77,78]. Comprehensive healthcare treatments will reduce payments for service delivery, especially for poor older adults with multi-morbidity, and improve health outcomes and quality of life. Considering the long-term healthcare outcomes in the older population, chronic care services are recommended. To better examine older adults’ patterns of use of healthcare services, future studies need to include primary care service utilization among older adults with multi-morbid health conditions. Given the inconsistent results on aging baby boomers’ health conditions [79] and their help-seeking attitudes [80], future research exploring the age cohort effect on older adults’ health profiles will be important. 

## Figures and Tables

**Table 1:** LCA model comparisons by the model fit indices (*n* = 6325).

	Class 2		Class 3		Class 4		Class 5	
BIC	27,868.128		27,789.460		27,736.929		27,743.495	
Entropy	0.86		0.92		0.76		0.79	
LMR LR test (*p* value)	<0.001		<0.001		0.001		0.004	
Class Counts and Proportions	740	0.12	671	0.11	251	0.04	172	0.03
	5585	0.88	671	0.11	5190	0.82	356	0.06
			1004	0.16	522	0.08	5183	0.82
					362	0.06	253	0.04
							361	0.06

**Table 2:** Multinomial logistic regression results on health condition profiles (*n* = 6294).

	Physical Only			Physical and Mental			Behavioral		
	RRR	95% Cl	*p*	RRR	95% Cl	*p*	RRR	95% Cl	*p*
Age group (ref: 50–64)									
65 and over	0.26	[0.19, 0.37]	<0.001	0.41	[0.33, 0.51]	<0.001	1.48	[1.17, 1.87]	0.001
Race (ref: White)									
Black	0.18	[0.09, 0.37]	<0.001	0.76	[0.56, 1.03]	0.076	1.61	[1.18, 2.18]	0.002
Hispanic	0.91	[0.59, 1.42]	0.685	0.75	[0.53, 1.06]	0.109	1.15	[0.80, 1.66]	0.456
Other	1.24	[0.69, 2.24]	0.468	1.59	[1.04, 2.42]	0.030	1.63	[0.99, 2.70]	0.056
Gender (ref: Male)									
Female	1.83	[1.38, 2.41]	<0.001	1.34	[1.11, 1.62]	0.003	1.07	[0.85, 1.33]	0.573
Marital status (ref: Others)									
Married	0.40	[0.29, 0.56]	<0.001	0.48	[0.38, 0.62]	<0.001	0.64	[0.49, 0.84]	0.001
Education (ref: Less than high school)									
High school	0.83	[0.54, 1.27]	0.388	0.75	[0.57, 0.99]	0.045	0.62	[0.46, 0.84]	0.002
College	1.01	[0.65, 1.55]	0.978	0.60	[0.44, 0.81]	0.001	0.70	[0.51, 0.96]	0.029
Above college	0.78	[0.49, 1.23]	0.282	0.60	[0.44, 0.82]	0.002	0.33	[0.22, 0.48]	<0.001
Insurance (ref: Not covered)									
Covered	0.39	[0.23, 0.65]	<0.001	0.69	[0.51, 0.96]	0.025	0.90	[0.60, 1.35]	0.605
Poverty (ref: Not in poverty)									
In poverty	3.15	[2.19, 4.52]	<0.001	2.10	[1.16, 2.74]	<0.001	1.69	[1.23, 2.30]	0.001
Household composition (ref: Living alone)									
Living with other family members	1.49	[1.05, 2.12]	0.025	1.02	[0.80, 1.31]	0.864	1.43	[1.06, 1.95]	0.020
Constant	0.73	[0.08, 0.36]		0.31	[0.19, 0.51]		0.09	[0.05, 0.16]	

Reference group: Healthy. RRR stands for Relative Risk Ratio; CI stands for confidence interval.

**Table 3:** Multinomial logistic regression results of the main and interaction effects of health profiles and poverty on specialty healthcare service use (*n* = 6278).

	Mental Health Treatment			Substance Use Treatment			Both Treatments		
	RRR	95% Cl	*p*	RRR	95% Cl	*p*	RRR	95% Cl	*p*
Health condition profiles (ref: Healthy)									
Physical and mental health problem	88.55	[53.37, 146.9]	<0.001	3.01	[0.87, 10.47]	0.083	249.41	[118.4, 525.3]	<0.001
Behavioral health problem	13.43	[10.42, 17.33]	<0.001	3.56	[2.30, 5.52]	<0.001	40.42	[22.94, 71.24]	<0.001
Physical health problem	3.21	[2.27, 4.53]	<0.001	1.08	[0.57, 2.05]	0.815	5.79	[2.30, 14.59]	<0.001
Age group (ref: 50–64)									
65 and over	0.53	[0.44, 0.64]	<0.001	0.42	[0.31, 0.57]	<0.001	0.43	[0.26, 0.71]	0.001
Race (ref: White)									
Black	0.39	[0.28, 0.55]	<0.001	1.03	[0.71, 1.50]	0.872	1.01	[0.56, 1.83]	0.970
Hispanic	0.35	[0.24, 0.52]	<0.001	0.69	[0.42, 1.14]	0.146	0.69	[0.35, 1.36]	0.286
Other	0.55	[0.44, 0.64]	0.015	1.22	[0.66, 2.24]	0.523	1.50	[0.70, 3.21]	0.294
Gender (ref: Male)									
Female	1.85	[1.54, 2.22]	<0.001	0.24	[0.18, 0.32]	<0.001	0.71	[0.48, 1.04]	0.081
Marital status (ref: Others)									
Married	0.96	[0.76, 1.22]	0.738	0.42	[0.30, 0.58]	<0.001	0.79	[0.48, 1.31]	0.364
Education (ref: Less than high school)									
High school	1.04	[0.77, 1.41]	0.807	1.03	[0.70, 1.51]	0.899	0.84	[0.46, 1.54]	0.581
College	1.30	[0.95, 1.78]	0.102	0.99	[0.66, 1.49]	0.959	1.62	[0.90, 2.92]	0.111
Above college	1.80	[1.31, 2.46]	<0.001	0.59	[0.37, 0.93]	0.023	1.25	[0.65, 2.41]	0.504
Insurance (ref: Not covered)									
Covered	0.47	[0.32, 0.68]	<0.001	0.77	[0.51, 1.16]	0.203	0.74	[0.39, 1.39]	0.349
Poverty (ref: Not in poverty)									
In poverty	1.62	[1.08, 2.42]	0.020	1.31	[0.85, 2.03]	0.219	3.86	[1.62, 9.19]	0.002
Household composition (ref: Living alone)									
Living with other family members	0.79	[0.61, 1.02]	0.077	0.78	[0.56, 1.09]	0.151	0.56	[0.34, 0.92]	0.022
Interaction effect (ref: Not in poverty × healthy group)									
Poverty × Physical and mental health problem	0.46	[0.17, 1.24]	0.126	-	-	1.000	0.20	[0.05, 0.80]	0.023
Poverty × Behavior health problem	0.66	[0.35, 1.23]	0.194	0.65	[0.26, 1.61]	0.351	0.31	[0.11, 0.93]	0.037
Poverty × Physical health problem	0.33	[0.12, 0.90]	0.030	0.66	[0,16, 2.67]	0.559	0.13	[0.01, 1.31]	0.083
Constant	0.18	[0.10, 0.30]		0.31	[0.16, 0.59]		0.02	[0.01, 0.05]	

Reference group: No treatment. RRR stands for relative risk ratio; CI stands for confidence interval.
